# Editorial: Technology aided personalized motor rehabilitation for individuals with neurological diseases

**DOI:** 10.3389/fbioe.2025.1595330

**Published:** 2025-03-27

**Authors:** Qinyin Qiu, Kiran K. Karunakaran, Hannah Aura Shoval, Ahmad O. Alokaily

**Affiliations:** ^1^ Department of Rehabilitation and Movement Sciences, School of Health Professions, Rutgers University, Newark, NJ, United States; ^2^ Center for Mobility and Rehabilitation Engineering Research, Kessler Foundation, West Orange, NJ, United States; ^3^ New Jersey Medical School, Rutgers University, Newark, NJ, United States; ^4^ Pediatric Physical Medicine and Rehabilitation, Atlantic Health System, Morristown, NJ, United States; ^5^ Department of Biomedical Technology, College of Applied Medical Sciences, King Saud University, Riyadh, Saudi Arabia

**Keywords:** neuro rehabilitation, rehabilitation technology, neurological diseases, personalize rehablitation, motor recovery

Personalized motor rehabilitation is the use of individualized interventions that are tailored to each person’s deficits and recovery progression. Technology-supported personalized motor rehabilitation takes this approach one step further by using technology to quantitatively determine deficits and inform treatment decisions, resulting in even more precisely customized and effective rehabilitation. It allows for adjustments to the treatment plan, tracking progress, and providing feedback to users, which can help with motivation.

Recently, there has been an increasing focus on using technology to support personalized motor rehabilitation. By leveraging advanced tools such as robotics, virtual reality (VR), wearable sensors, machine learning, and neuromodulation, researchers and clinicians can now design interventions tailored to each patient’s unique needs. This Research Topic, *“Technology-Aided Personalized Motor Rehabilitation for Individuals with Neurological Diseases,”* brings together a diverse Research Topic of studies that explore innovative technologies, their applications, and the challenges of implementing them in clinical practice. The articles in this Research Topic highlight the potential of these approaches to improve outcomes while addressing the challenges of accessibility, affordability, and clinician training.

One of the key themes in this Research Topic is the development and validation of new technology-based interventions. Harrington et al. explored the use of aquatic treadmill walking to compare muscle co-contraction in children with cerebral palsy and typically developing children. The authors examined how different calculation methods could alter clinical interpretations, underscoring the importance of standardized metrics and robust analytical frameworks. Such work highlights the influence of data analysis choices on treatment decisions, reinforcing the need for evidence-based, quantitative methodologies to guide clinicians. Similarly, Cerfoglio et al. demonstrated the potential of low-cost solutions like the Azure Kinect to compare gait parameters in healthy and hemiplegic individuals. Their findings suggest that single-camera systems may offer viable alternatives for assessing patient progress in less resource-intensive settings. This line of research speaks to the potential of portable and more affordable solutions, particularly relevant for telehealth or remote rehabilitation contexts when in-person evaluations are not feasible.

Virtual reality also plays a prominent role in this Research Topic. Beyond gait, Bonanno et al. examined whether the Computer Assisted Rehabilitation ENvironment (CAREN)—including a split-belt treadmill, six-degrees-of-freedom motion platform, and large-scale VR display—could benefit patients with cerebellar ataxia. Their pilot study suggests that such immersive VR-based interventions may yield improvements in balance and gait mechanics, contributing to the growing body of evidence supporting VR’s capacity to provide task-oriented and engaging therapy. Berger and d’Avella took a different approach by combining myoelectric control with VR to enhance upper-limb rehabilitation for people with stroke. By providing personalized feedback based on real-time electromyographic signals, their study demonstrates how VR can be used to re-train dysfunctional muscle activation patterns and facilitate motor recovery - a prime example of how individualized feedback can re-engage neuroplastic processes.

Neuromodulation is another promising avenue explored in this Research Topic. Zhang et al. studied non-invasive cervical spinal cord neuromodulation via trans-spinal electrical stimulation (tsES) on cortico-muscular coherence in people with stroke. Their findings highlighted how tsES can modulate cortico-muscular coherence, potentially enhancing descending excitatory control and inhibiting unwanted compensatory mechanisms in chronic stroke. This form of personalized neuromodulation could be integrated with other therapies to optimize motor recovery strategies.

Huang et al. was able to identify mores subtle proprioception deficits in the common condition of knee osteoarthritis by using the technological assistance of the ankle inversion discrimination apparatus for walking (AIDAW). Their findings emphasized that deficits in lower-limb proprioception may increase the risk of falls among individuals with knee osteoarthritis. This research points to the importance of evaluating multiple biomechanical and sensory factors in rehabilitation plans, reinforcing that personalization must account for not just gross mechanical deficits affecting movement parameters but also neurologic factors such as somatosensory function.

Despite the promise of these technologies, challenges remain. Albishi et al. surveyed the acceptance and level of knowledge about transcranial magnetic stimulation (TMS) among Saudi Arabia’s rehabilitation specialists. Their survey underscores that technology alone is insufficient; systematic education and awareness are essential to ensuring clinicians can effectively adopt and apply emerging interventions. These findings resonate with the broader challenge of working at the crossing point of technology and clinical practice, where training, regulatory frameworks, and interdisciplinary cooperation are crucial for safe and efficacious implementation.

The articles in this Research Topic collectively emphasize the transformative potential of technology-aided personalized motor rehabilitation. However, they also highlight the need for interdisciplinary collaboration to address key challenges, including standardization, accessibility, and clinician training. To realize the full potential of this field, further research is needed to (1) standardize and validate novel assessments, (2) develop robust algorithms capable of real-time adaptation to individual patient needs, (3) ensure affordability and portability of devices, and (4) incorporate user feedback to maintain motivation and therapy adherence. Ultimately, interdisciplinary collaboration between researchers, healthcare providers, technology developers, and regulatory bodies, drive these advances forward—from engineers refining device designs, to clinicians interpreting new data and personalizing therapy, to policymakers fostering frameworks that encourage widespread adoption.

As we look to the future, it is essential to continue exploring the role of machine learning and artificial intelligence in personalizing rehabilitation and investigating patient and caregiver perspectives to ensure that these technologies are user-friendly and effective. By addressing these challenges, we can unlock the full potential of technology-supported personalized motor rehabilitation, improving outcomes and quality of life for individuals with neurological diseases.

